# Human epidermal neural crest stem cells as a source of Schwann cells

**DOI:** 10.1242/dev.123034

**Published:** 2015-09-15

**Authors:** Motoharu Sakaue, Maya Sieber-Blum

**Affiliations:** Institute of Genetic Medicine, Newcastle University, Centre for Life, Newcastle upon Tyne NE1 3BZ, UK

**Keywords:** hEPI-NCSC, Neural crest, Schwann cell, Peripheral nerve, SOX10, KROX20, Protein zero, EPI-NCSCs

## Abstract

We show that highly pure populations of human Schwann cells can be derived rapidly and in a straightforward way, without the need for genetic manipulation, from human epidermal neural crest stem cells [hEPI-NCSC(s)] present in the bulge of hair follicles. These human Schwann cells promise to be a useful tool for cell-based therapies, disease modelling and drug discovery. Schwann cells are glia that support axons of peripheral nerves and are direct descendants of the embryonic neural crest. Peripheral nerves are damaged in various conditions, including through trauma or tumour-related surgery, and Schwann cells are required for their repair and regeneration. Schwann cells also promise to be useful for treating spinal cord injuries. *Ex vivo* expansion of hEPI-NCSC isolated from hair bulge explants, manipulating the WNT, sonic hedgehog and TGFβ signalling pathways, and exposure of the cells to pertinent growth factors led to the expression of the Schwann cell markers SOX10, KROX20 (EGR2), p75NTR (NGFR), MBP and S100B by day 4 in virtually all cells, and maturation was completed by 2 weeks of differentiation. Gene expression profiling demonstrated expression of transcripts for neurotrophic and angiogenic factors, as well as JUN, all of which are essential for nerve regeneration. Co-culture of hEPI-NCSC-derived human Schwann cells with rodent dorsal root ganglia showed interaction of the Schwann cells with axons, providing evidence of Schwann cell functionality. We conclude that hEPI-NCSCs are a biologically relevant source for generating large and highly pure populations of human Schwann cells.

## Abstract (precis)

**Summary:** Human epidermal neural crest stem cells isolated from the bulge of hair follicles are used to derive Schwann cells that could be useful for regenerative therapies, disease modelling and drug discovery.

## INTRODUCTION

Schwann cells are neural crest-derived glia that support axons of peripheral nerves. Injuries to peripheral nerves are common; they can be due to neuropathies, traumatic injury, tumour-related surgery or repetitive compression (Pfister et al., 2011; Griffin et al., 2014; Evans, 2001). Overall, peripheral nerve injury carries a high cost to healthcare systems (Rosberg et al., 2005; Noble et al., 1998). After peripheral nerve injury, the proximal stump of the nerve is capable of regeneration and reinnervation. Surgical repair for minor nerve reconstruction involves direct end-to-end anastomosis (Griffin et al., 2014). In significant nerve injuries, nerve repair requires bridging the gap and the introduction of Schwann cells, together with Schwann cell-derived growth factors and an extracellular matrix for guiding axonal extension and nerve regeneration (Shirosaki et al., 2014). Axons regenerate along aligned Schwann cells called bands of Büngner, which form a permissive environment (Allodi et al., 2012). Scaffolds and tubes are used as guidance materials for nerve grafts. Currently, autografts from sensory nerves are typically used to repair large peripheral nerve damage. Autografts have several disadvantages, however, including damage to the donor nerve caused by the biopsy. Schwann cells have also proven useful to treat spinal cord injuries (e.g. Wiliams and Bunge, 2012; Bunge, 2008). The availability of large numbers of human Schwann cells for autologous use and for disease modelling and drug discovery is highly desirable.

Here, we provide a methodology for using hEPI-NCSC to generate large and highly pure populations of human Schwann cells using a straightforward strategy that avoids genetic modification and includes manipulation of pertinent signalling pathways with small molecules. hEPI-NCSC are multipotent adult stem cells that derive from the neural crest, a transient tissue in vertebrate embryos that originates from the dorsal aspect of the developing neural tube, the future spinal cord. Neural crest cells become migratory and translocate away from the forming neural tube to various locations within the embryo, where they give rise to a wide array of cell types and tissues, including the Schwann cells of the peripheral nervous system (Le Douarin and Kalcheim, 1999). A subset of multipotent embryonic neural crest cells invade the ectoderm early in development (Richardson and Sieber-Blum, 1993; Narytnyk et al., 2014a), some of which become located in a stem cell niche of the hair follicle, the ‘bulge’, where they persist postnatally and into adulthood (Sieber-Blum et al., 2004; Sieber-Blum and Grim, 2004; Hu et al., 2006; Clewes et al., 2011; Sieber-Blum, 2014). Another type of neural crest-derived skin progenitor cell is a Schwann cell precursor that arrives in the skin via projecting nerves and gives rise to melanocytes (Adameyko et al., 2009). Cultured hEPI-NCSC proliferate rapidly, generating millions of stem cells within a short period of time (Clewes et al., 2011), and they can undergo directed differentiation into various cell types of clinical relevance, including midbrain dopaminergic neurons (Narytnyk et al., 2014b), osteocytes and melanocytes (Clewes et al., 2011). In mouse models of spinal cord injury, murine EPI-NCSC have shown efficacy by eliciting significant improvements in sensory connectivity and touch perception (Sieber-Blum et al., 2006; Hu et al., 2010), and canine EPI-NCSC (cEPI-NCSC) are promising candidates for treating spinal cord injuries in dogs (Gericota et al., 2014; McMahill et al., 2014; McMahill et al., 2015).

Various types of stem cell have been described as being able to differentiate into Schwann-like cells and in assisting peripheral nerve regeneration, primarily adipose tissue-derived stem cells (Kingham et al., 2007; Reid et al., 2011; Tomita et al., 2013; Kolar and Kingham, 2014; Razavi et al., 2013), as well as human umbilical cord-derived mesenchymal stem cells (Lee et al., 2011), skin mesenchymal precursors (Krause et al., 2014), embryonic stem cell-derived neural crest cells (Ren et al., 2013), human amniotic membrane (Banerjee et al., 2014), mesenchymal stem cells (Pan and Cai, 2012), amniotic mesenchymal stem cells (Jiang et al., 2010) and human embryonic stem cell-derived neurospheres (Ziegler et al., 2011).

The primary significance of hEPI-NCSC is that they are biologically the most relevant cell type to generate Schwann cells, as they are direct descendants of the embryonic neural crest, which is the source of Schwann cells in the body. We show here that highly pure populations of human Schwann cells can be generated from *ex vivo* expanded hEPI-NCSC rapidly and with high efficiency. There is no need for purification because, by taking advantage of the migratory ability of neural crest cells, highly pure populations of hEPI-NCSC are generated in primary culture. Notably, hEPI-NCSC can be isolated by a minimally invasive procedure via a small biopsy of hairy skin and they can be expanded *ex vivo* into millions of stem cells in adherent culture (Clewes et al., 2011). Furthermore, hEPI-NCSC-derived Schwann cells express neurotrophins and other factors essential for nerve regeneration. Similar to mouse EPI-NCSC (mEPI-NCSC; GEO accession number GSE4680; Hu et al., 2006; Sieber-Blum et al., 2006) and cEPI-NCSC (McMahill et al., 2014; McMahill et al., 2015), hEPI-NCSC and Schwann cells derived therefrom express the *VEGFA* and *VEGFB* genes (GEO accession number GSE61273). This is an important aspect, as angiogenesis is crucial for nerve repair (Kolar and Kingham, 2014). Importantly, as we have shown in the mouse spinal cord (Hu et al., 2010), in canine spinal cord (McMahill et al., 2015), in athymic rats (M.S.-B., unpublished data) and in a teratoma assay (McMahill et al., 2015), EPI-NCSC do not form tumours *in vivo*, which is a hallmark of adult stem cells.

*In vivo*, Schwann cell precursors differentiate into immature Schwann cells, which subsequently undergo terminal differentiation into one of two types of mature Schwann cell: myelinating and non-myelinating. Myelinating Schwann cells myelinate large diameter neurons, whereas non-myelinating Schwann cells (Remak cells) form mesaxons with unmyelinated small diameter neurons. Upon nerve injury, Schwann cells alter their characteristics to acquire a Schwann repair cell phenotype (Jessen and Mirsky, 2005; Arthur-Farraj et al., 2012). Schwann cell differentiation and maintenance are dependent on growth factors that are provided by the embryonic microenvironment and the axons that they interact with (Jessen and Mirsky, 1999, 2005). The experimental design in this study is based on these growth factor requirements.

## RESULTS

### Experimental design and master gene regulation

**Fig. 1.**
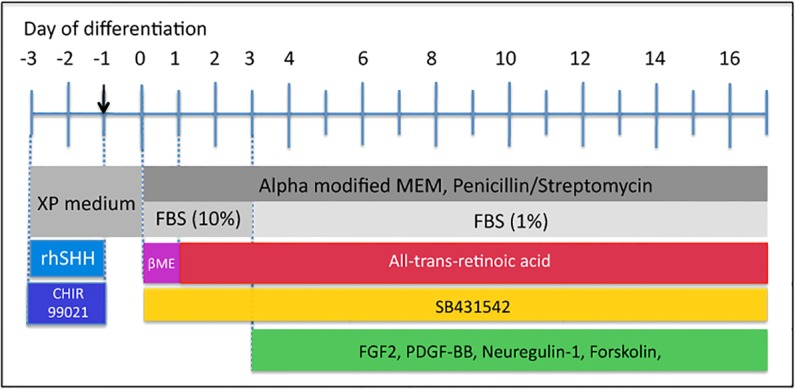
**Experimental design.** Cells were pretreated with rhSHH and CHIR99021 for 24 h in expansion (XP) medium [differentiation day (D) −3 to D−1]. Cells were then subcultured on D−1 (arrow) into XP medium and allowed to recover for 24 h. On D0, SB431542 was first added and cells were treated with β-mercaptoethanol (βME) for 24 h. On D1, retinoic acid (RA) was first added. On D3, differentiation factors FGF2, PDGF-BB, NRG1 and forskolin were first added. All components were added fresh every 48 h.

SOX10 function plays central roles in Schwann cell fate decision and differentiation (Kelsh, 2006). hEPI-NCSC expressed SOX10, as assessed by immunoreactivity, but predominantly in the cytoplasm (data not shown). Pilot experiments suggested that this SOX10 expression pattern was not conducive to efficient Schwann cell differentiation. As SHH signalling and WNT signalling regulate SOX10 expression (McNeill et al., 2012; Werner et al., 2007), cells were treated transiently with SHH and the GSK3β inhibitor CHIR99021, which led to SOX10 localisation in the nucleus and to highly efficient Schwann cell differentiation (Fig. 1).

On day 0 of differentiation (D0), cells were treated for 24 h with β-mercaptoethanol (βME), and all-trans retinoic acid (RA) was added on D1 according to the method of Dezawa et al. (2001). However, neither 3 nor 5 days of RA treatment was sufficient for Schwann cell differentiation, as judged by cell morphology and marker expression. RA was therefore left in the differentiation medium throughout the culture period without any detectable deleterious effects on the cells. At D0, SB431542, an inhibitor of transforming growth factor beta 1 (TGFβ1) activin receptor-like kinases (ALKs), was also added to the culture medium (Fig. 1). The continued presence of SB431542 in the culture medium was essential. On D3, FGF2, PDGF-BB, forskolin and neuregulin 1 (NRG1) were added.

### Cell morphology during *in vitro* differentiation of hEPI-NCSC

**Fig. 2.**
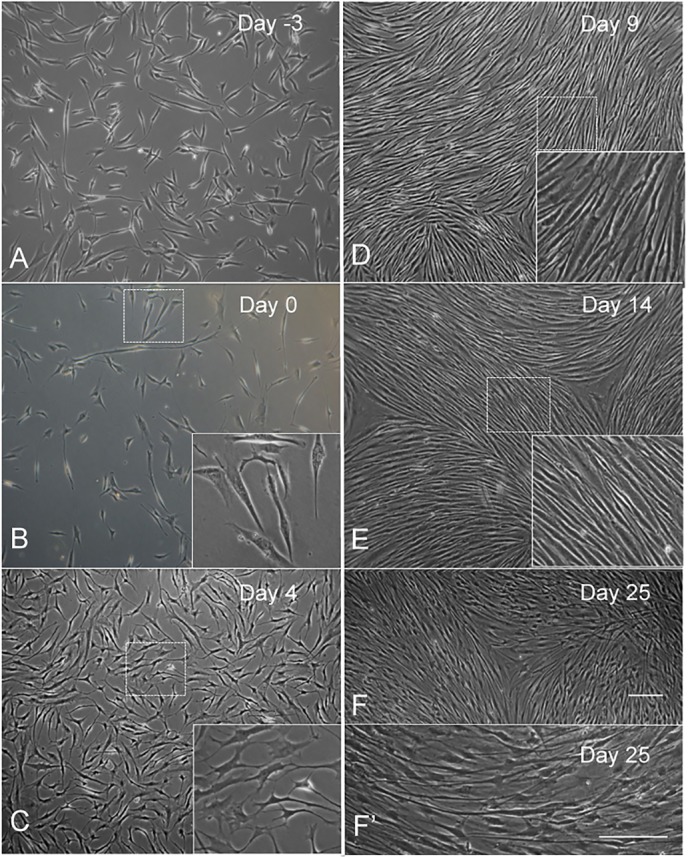
**Cell morphology before and during differentiation.** (A) D−3, showing stellate morphology typical for neural crest cells. (B) D0, showing unchanged cell morphology after SHH and CHIR99021 treatment. (C) D4, cells continued to proliferate and started to change morphology. (D-F) D9 and later, cells became elongated and morphology was maintained in prolonged culture. F′ shows cells at higher magnification. Scale bars: 50 µm.

Prior to differentiation, hEPI-NCSC had the typical stellate morphology of neural crest stem cells (Fig. 2A), which remained unchanged after pretreatment with SHH and CHIR99021 and subculture (Fig. 2B). By D4, cells became more elongated (Fig. 2C). By D9, cells had assumed the slender, elongated morphology of Schwann cells and started to form swirls in the culture plate (Fig. 2D); they maintained this morphology for as long as they were kept in culture (up to 30 days; Fig. 2E,F). Under these conditions, cells continued to proliferate in differentiation culture until approximately D9-D14. Schwann cells could be cryopreserved and were viable after thawing and reculturing.

### Timecourse of Schwann cell marker expression

**Fig. 3.**
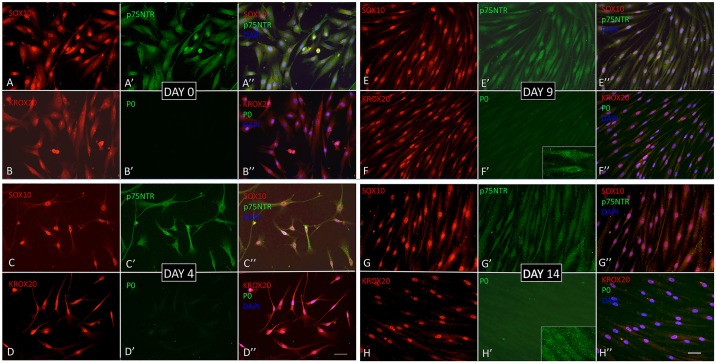
**Immunocytochemistry.** (A-B″) D0. (A-A″) All cells exhibit SOX10 (A) and p75NTR (A′) immunofluorescence. (B-B″) KROX20 is expressed in all cells (B), whereas P0 is undetectable (B′). (C-D″) D4. (C-C″) All cells exhibit SOX10 (C) and p75NTR (C′) immunofluorescence. (D-D″) All cells exhibit KROX20 immunoreactivity (D), while P0 immunoreactivity is detected in all cells at low levels (D′). (E-F″) D9. (E-E″) All cells are SOX10 positive, showing intense SOX10 nuclear immunoreactivity (E), but decreased p75NTR immunoreactivity (E′). (F-F″) All cells show strong KROX20 immunoreactivity, mostly nuclear (F), and weak P0 immunoreactivity (F′); inset shows P0 immunoreactivity at higher magnification. (G-H″) D14. (G-G″) Cells show strong nuclear SOX10 immunoreactivity (G) and weak p75NTR immunoreactivity (G′). (H-H″) KROX20 immunoreactivity, showing mostly nuclear localisation (H), and weak P0 immunoreactivity in all cells (H′); inset shows P0 immunoreactivity at higher magnification. DAPI is blue in merged images (A″-H″). Scale bars: 50 µm.

Robust Schwann cell marker expression was observed by indirect immunocytochemistry. All cells were immunopositive for the neural crest stem cell and Schwann cell marker SOX10 (Table 1). Nuclear SOX10 immunoreactivity was observed in increasing numbers of cells with progressing differentiation, with a maximum of 95.4±1.4% by D4, persisting until D14 (89.0±2.5%) and subsequently declining (Fig. 3, Table 1; supplementary material Fig. S1). KROX20 (EGR2) is a key marker for myelinating Schwann cells and is regulated by SOX10 (Jessen and Mirsky, 2002; Reiprich et al., 2010) and RA (Heinen et al., 2013). All cells expressed KROX20. Nuclear expression of KROX20 was observed in increasing numbers of cells, with 91.9±0.8% on D9, increasing to a maximum of 95.6±1.2% by D14 and, in contrast to SOX10, without any significant decline thereafter (Fig. 3, Table 1; supplementary material Fig. S1). All cells expressed p75NTR (NGFR; a neural crest stem cell maker), myelin basic protein (MBP) and S100B, as assessed by immunoreactivity, throughout the culture period. The intensity of p75NTR immunofluorescence visibly decreased with progressing cell differentiation (Fig. 3, Table 1; supplementary material Figs S1 and S2). By contrast, glial fibrillary acidic protein (GFAP) immunoreactivity was not detected initially, and was at barely detectable levels only by D30 (supplementary material Fig. S2; Table 1). Cells were, however, intensely GFAP-immunoreactive in the absence of RA, SHH and CHIR99021, with predominantly cytoplasmic SOX10 expression (supplementary material Fig. S3). Myelin P-zero (P0) immunoreactivity was not detectable initially, became detectable at D4, increased in intensity thereafter and remained strong throughout the remainder of the culture period (Fig. 3, Table 1; supplementary material Fig. S1). Marker expression was confirmed at the RNA level by qPCR (Table 2).

**Table 1.**
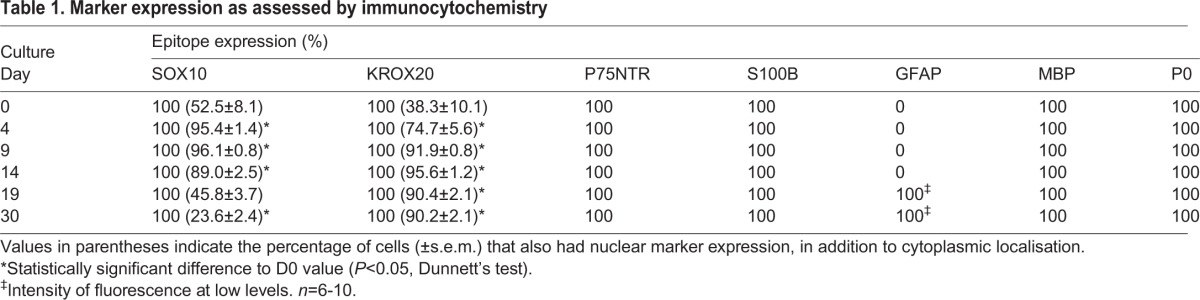
**Marker expression as assessed by immunocytochemistry**

**Table 2.**
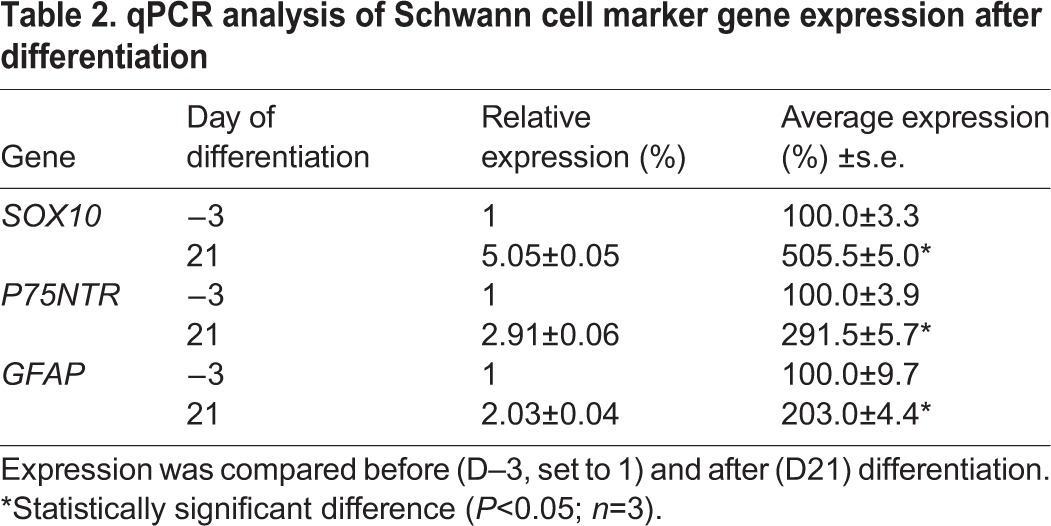
**qPCR analysis of Schwann cell marker gene expression after differentiation**

### Differential gene expression in hEPI-NCSC-derived Schwann cells

Two comprehensive gene expression profiles were generated using the Illumina platform and an HT12 v4 Array (GEO accession number GSE61273), one with RNA from undifferentiated hEPI-NCSC and the other with RNA from hEPI-NCSC-derived Schwann cells. The Diff Score is a transformation of the *P*-value that provides directionality to the *P*-value based on the difference between the average signal in the reference group versus the comparison group. Of a total of 47,229 transcripts, 3690 genes were differentially expressed with *P*≤0.01; of these, 2954 genes were differentially expressed with *P*≤0.001.

As expected, highly differentially expressed genes concerned primarily metabolic processes, general differentiation markers, transporters of molecules, and regulation of cell behaviour. Thirty-one significantly downregulated transcripts in Schwann cells (*P*≤0.01) concerned cell migration, and an estimated >200 downregulated genes (*P*<0.01) regulate cell proliferation. Extracellular proteases produced by migrating neural crest cells serve to degrade embryonic extracellular matrix. Four metalloproteases of the MMP family [*MMP2*, *MMP3*, *IMP2* (*IMMP2L*) and *BSG*; *P*<0.0001] and nine members of the ADAM family of proteinases (*ADAM12*, *15*, *17*; and *ADAMTS2*, *5*, *6*; *ADAMTSL1*, *4*; *P*<0.001) were significantly downregulated.

Transcripts of *KROX20*, a gene specific for the myelinating type of Schwann cell, were upregulated 1.45-fold (*P*<0.001) in Schwann cells. Several myelin proteins were upregulated. *MBP* transcripts, for instance, were upregulated 3.2-fold (*P*<0.001). Cholesterol is a major component of myelin. Twenty-one genes involved in cholesterol metabolism were upregulated with high significance. For example, transcripts encoding MALL, a member of the MAL family of proteolipids that localises to cholesterol-enriched membranes, were upregulated 3.4-fold (*P*<0.001). Emopamil-binding protein (*EBP*), which was upregulated 1.7-fold (*P*<0.001), encodes a sterol isomerase involved in synthesizing 3β-hydroxysteroid-Δ8,Δ7-isomerase, which in turn is responsible for one of the final steps in the production of cholesterol. Transcripts encoding OSBPL5, a member of the oxysterol-binding protein (OSBP) family, which is a group of intracellular lipid receptors that play a key role in maintaining cholesterol balance in the body, were upregulated 1.14-fold (*P*<0.001). Transcripts of superoxide dismutase 1 (*SOD1*), mutations in which are implicated in multiple sclerosis, a de-myelinating disease, were upregulated 1.7-fold (*P*<0.001) in Schwann cells. Transcripts of caveolin 1 (*CAV1*), mutations in which are associated with Berardinelli–Seip congenital lipodystrophy, were upregulated 2.2-fold (*P*<0.001) (Table 3).

**Table 3.**
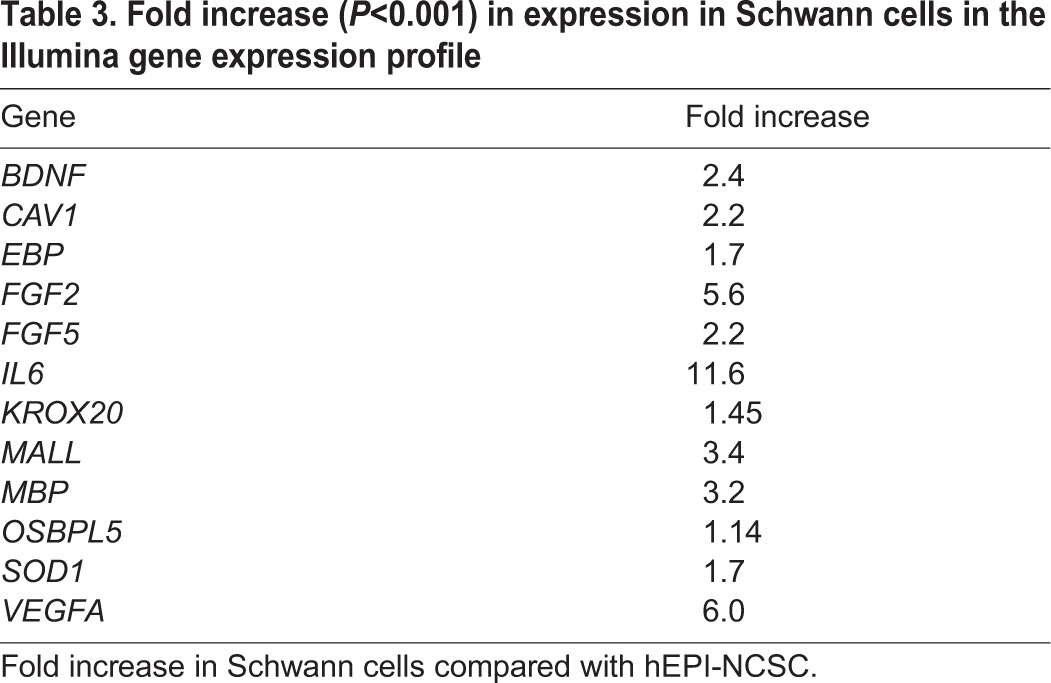
**Fold increase (*P*<0.001) in expression in Schwann cells in the Illumina gene expression profile**

*In vivo*, Schwann cells produce neurotrophins and other factors that support neuronal survival, including nerve growth factor (NGF) (Rush, 1984), neurotrophin 4/5 (NT-4/5; NTF4) (Funakoshi et al., 1993), brain-derived neurotrophic factor (BDNF) (Meyer et al., 1992), fibroblast growth factor 2 (FGF2) (Ornitz and Itoh, 2001), FGF5 (McGeachie et al., 2001), leukaemia inhibitory factor (LIF) (Kurek et al., 1996) and interleukin 6 (IL6) (Bolin et al., 1995). Neurotrophin 3 (*NT3*; *NTF3*), *FGF2*, *BDNF* and *LIF* were expressed in both hEPI-NCSC and Schwann cells derived therefrom with highest probability (detection *P*=0). *FGF5* (2.2-fold), *BDNF* (2.4-fold), *FGF2* (5.6-fold) and *IL6* (11.6-fold) were significantly upregulated in Schwann cells (Table 3). Angiogenesis is an important aspect of successful nerve regeneration. Both *VEGFA* and *VEGFB* were expressed in hEPI-NCSC and in Schwann cells derived therefrom with highest probability (detection *P*=0); *VEGFA* was upregulated sixfold (Table 3). JUN (c-Jun) expression in Schwann cells is essential and sufficient for supporting nerve repair, as it regulates the de-differentiation of myelinating Schwann cells into the regenerative type of Schwann cell (Arthur-Farraj et al., 2012). *JUN* transcripts were detected in both hEPI-NCSC and hEPI-NCSC-derived Schwann cells with highest probability (detection *P*=0). See also the heat map in supplementary material Fig. S4.

### Functionality of hEPI-NCSC-derived human Schwann cells *in vitro*

**Fig. 4.**
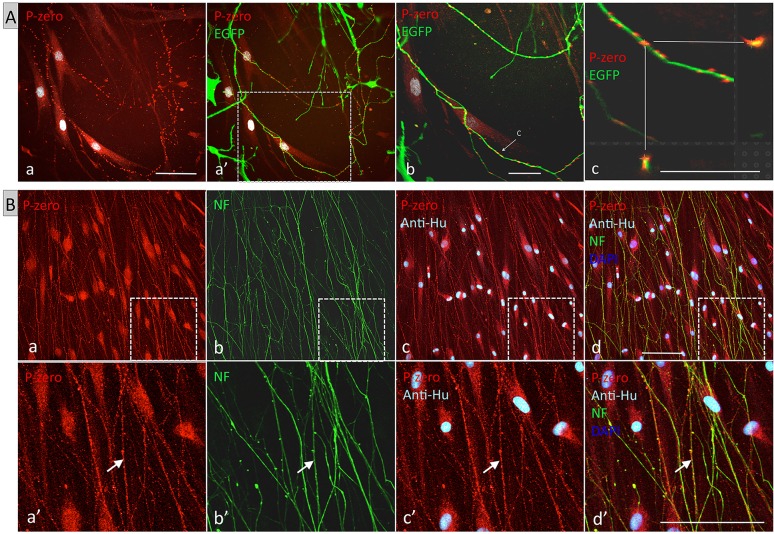
**Interaction of hEPI-NCSC-derived Schwann cells with neurites from rodent DRG as observed by confocal microscopy.** (A) Cultures fixed at 11 days of co-culture with dissociated DRG neurons from adult ‘Green’ mouse (EGFP fluorescence). (a) P0 immunoreactivity (red) and anti-human nuclear antibody (pale blue) merged images. Four human Schwann cells are visible; bead-like P0 immunofluorescence is apparent above the Schwann cells. (a′) Merge of area in panel a with EGFP fluorescence. The bead-like P0-positive structures (red) correspond precisely with neuronal processes (green). (b) The boxed area in a′ at higher magnification. Slice thickness is 0.72 µm. (c) Orthogonal reconstruction of the area indicated by the arrow in b, showing close interaction between Schwann cell process and neurite. (B) Cultures were fixed at day 21 of co-culture with whole DRG explants from E15.5 rat embryos. (a) P0 (red) fluorescence. Schwann cell bodies are in aligned orientation; bead-like structures are apparent in processes and on cell bodies. (b) Neurofilament fluorescence (green) in same area as in panel a. The orientation of neurites corresponds to that of Schwann cells in panel a. (c) P0 and anti-human nuclear antibody (anti-Hu, pale blue) merged images; all Schwann cells are of human origin. (d) Merge of P0, anti-human nuclear antibody, neurofilament and DAPI nuclear stain (dark blue) showing absence of any non-human cells. (a′-d′) Higher magnification of boxed areas in a-d; arrow indicates P0 and neurofilament double-positive structures. Scale bars: 50 µm.

Next we addressed the functionality of Schwann cells *in vitro* by determining whether hEPI-NCSC-derived Schwann cells can interact with neurites. We co-cultured hEPI-NCSC-derived Schwann cells with camptothecin-treated rodent dorsal root ganglia (DRG) explants (supplementary material Fig. S5). In contrast to cytosine arabinoside, in our hands (data not shown), camptothecin efficiently removed endogenous Schwann cells but was toxic selectively to large diameter sensory neurons (supplementary material Fig. S5A′), as has been documented previously (Bhanu and Kondapi, 2010). Confocal microscopy showed bead-like myelin P0 immunoreactivity above the human Schwann cells, which corresponded precisely with murine DRG neuronal processes (Fig. 4Aa,a′). Anti-human nuclear antibody immunofluorescence confirmed that all P0-immunoreactive Schwann cells were of human origin (Fig. 4Aa-b, pale blue fluorescence). There was a close interaction of Schwann cell-derived P0 immunoreactivity with neurite-derived EGFP fluorescence (Fig. 4Ac).

As E15.5 rat embryos do not yet have differentiated Schwann cells (Jessen et al., 1994), we repeated the co-culture experiments with whole DRG explants from E15.5 rat embryos. Whereas numerous emigrating non-neuronal cells were present in the absence of camptothecin (supplementary material Fig. S5B), camptothecin-treated DRG explants were clear of non-neuronal cells (supplementary material Fig. S5B′). hEPI-NCSC-derived Schwann cells aligned with the neurites radiating from the explant (Fig. 4B; supplementary material Fig. S5B″). The cell bodies and processes of Schwann cells were P0 immunoreactive (Fig. 4B). Again, bead-like Schwann cell P0 immunoreactivity was consistently and precisely in close association with neurites (Fig. 4B). All cells were immunopositive with the anti-human nuclear antibody (Fig. 4Bc), confirming the human origin of the Schwann cells. Anti-Hu/DAPI double staining showed that the co-cultures did not contain any non-human non-neural cells, as all DAPI-stained nuclei were also anti-Hu immunoreactive (Fig. 4Bd).

**Fig. 5.**
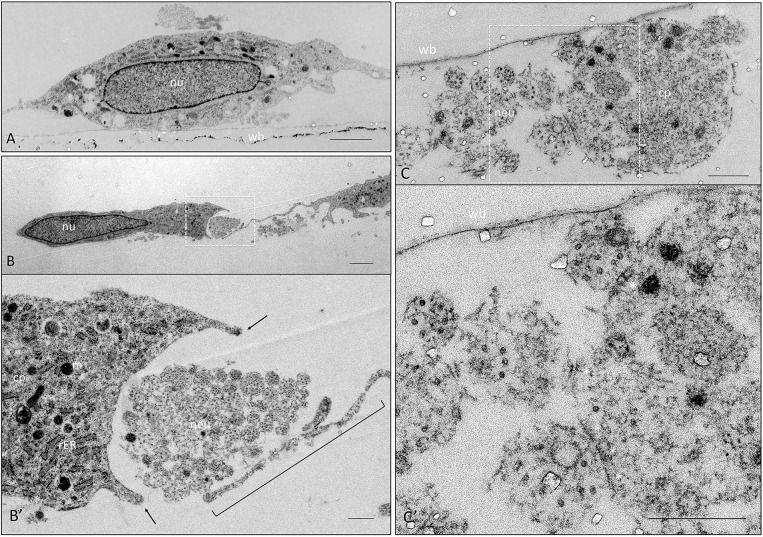
**Close interaction of hEPI-NCSC-derived Schwann cells with neurites from rodent DRG as observed by electron microscopy.** (A) A Schwann cell (nu, nucleus) attached to the well bottom (wb). In the cytoplasm, mitochondria (dark, rounded) and endoplasmic reticulum (elongated double-track structures) are visible. (B) Bundle of neurites in transverse section (boxed area) enclosed by processes from two cells: from the left (cell with visible nucleus) and a process from a cell to the right of the image. (B′) Higher magnification of boxed area in B. The cytoplasm (cp) of the cell to the left shows mitochondria (m) and endoplasmic reticulum (rER). Individual neurites (neu) within the cluster are visible in transverse section. Processes in proximity to the cluster of neurites are marked by arrows; the process originating from the cell to the right is indicated by a bracket. (C) Cluster of neurites in transverse section (microtubules detectable), showing three neurites in close membrane proximity with a cell to the right (the cytoplasm of which is visible). (C′) Higher magnification of boxed area in C. Three neurites have made close contact with the cell to the right (five asterisks). Note that the ultrastructure is compromised due to a long lag time between fixation and processing for electron microscopy, as well as shipping in buffer rather than in fixative. Scale bars: 5 µm in A,B; 500 nm in B′,C,C′.

Since experiments in two different co-culture systems showed close association of hEPI-NCSC-derived Schwann cells with neurites, we next investigated this interaction at the ultrastructural level in co-cultures of Schwann cells and rat embryo DRG. Fig. 5 shows electron microscopy images of hEPI-NCSC–DRG co-cultures. Fig. 5A shows a Schwann cell attached to the well bottom. Bundles of DRG-derived neurites were engulfed by protrusions from Schwann cells (Fig. 5B,B′, arrows and bracket). Fig. 5C,C′ show three neurites forming close membrane-to-membrane contacts with the cell on the right (marked by the five asterisks), which is considered a prerequisite for subsequent myelination. Thus, close interactions of hEPI-NCSC-derived Schwann cells with rat embryo DRG axons were also observed at the ultrastructural level.

## DISCUSSION

A readily available source of large numbers of highly pure human Schwann cells is desirable for use in surgery to repair damage to peripheral nerves, potentially for other cell-based therapies, and for research purposes. Here, we show that hEPI-NCSC are a biologically relevant source of adult stem cells for generating large and highly pure populations of human Schwann cells. hEPI-NCSC-derived Schwann cells exhibited characteristic morphology, expressed relevant markers at the protein and RNA levels, and interacted with axons of rodent DRGs in explant culture, providing evidence of the functionality of Schwann cells.

A central aspect of our methodology concerns the augmentation of SHH and canonical WNT signalling, which resulted in increased nuclear localisation of SOX10. The transcription factor SOX10 is a key regulator in neural crest formation and in Schwann cell fate decision and differentiation. SOX10 is also involved in regulating expression of the myelin proteins P0 and MBP. By contrast, SOX10 function must be shut off for neuronal differentiation to proceed (Rehberg et al., 2002). Although hEPI-NCSC are direct descendants of the embryonic neural crest, gene expression profiles of hEPI-NCSC and embryonic neural crest stem cells are not identical (Hu et al., 2006). Whereas SOX10 has a nuclear localisation in the embryonic neural crest, hEPI-NCSC showed predominantly cytoplasmic SOX10 immunoreactivity. WNT and SHH signalling are part of the network that regulates SOX10 expression (Werner et al., 2007; McNeill et al., 2012), which explains the increased nuclear SOX10 localisation in hEPI-NCSC after SHH and CHIR99021 treatment. SOX10 is a shuttle protein. Continuous entry and exit to and from the nucleus is essential for its function. This involves post-translational modification of SOX10 in the cytoplasm, which is lost over time while SOX10 resides in the nucleus (Rehberg et al., 2002). Nuclear entry/exit of SOX10 is thought to represent a fast-acting regulatory system for SOX10 function (Rehberg et al., 2002). SOX10 expression is nuclear *in vivo* in mature nerves and in distal stumps after nerve transection. The predominantly cytoplasmic localisation of SOX10 immunoreactivity at the end of the 30-day differentiation period might reflect a loss of the SHH and CHIR99021 effects.

SOX10 regulates the *KROX20* gene (Reiprich et al., 2010). Persistent KROX20 expression is characteristic of myelinating Schwann cells (Allodi et al., 2012), as is MBP and P0 expression. In the present study, Schwann cells exhibited strong nuclear KROX20 immunoreactivity throughout the culture period. Surprisingly, there was strong MBP expression already at D0. This can be explained by the fact that SOX10 and KROX20, both of which are expressed at that time, synergistically regulate MBP expression (Marathe et al., 2013). The intensity of MBP immunofluorescence declined somewhat with time in culture. However, in co-culture with DRG neurons we observed intense MBP immunofluorescence (data not shown), illustrating reciprocal influences between neurites and Schwann cells. p75NTR immunofluorescence was intense at the onset of differentiation of hEPI-NCSC into Schwann cells, due to the fact that p75NTR is expressed by neural crest stem cells. The intensity of p75NTR immunofluorescence decreased with progressing differentiation in culture, which is characteristic for myelinating Schwann cells. GFAP immunoreactivity was not detected initially but was present at modest levels by D30. However, in the absence of RA, SHH and CHIR99021, all cells were robustly GFAP immunoreactive in addition to showing cytoplasmic SOX10 localisation. These data suggested that the precise type, and possibly duration, of SOX10 function contributes to the identity of the Schwann cell subtype.

Gene expression profiling confirmed that with progressing *in vitro* differentiation Schwann cells reduced the expression of transcripts characteristic for neural crest stem cells, including genes involved in cell migration, cell proliferation and cell invasiveness. There was a concomitant increase in the expression of *MBP*, genes involved in cholesterol metabolism, which is relevant to myelin synthesis, and in the expression of transcripts of relevant neurotrophins and other pertinent growth factors. Importantly, gene expression profiling showed the expression of *JUN* transcripts, a transcription factor in Schwann cells that is essential for nerve repair as it regulates the molecular reprogramming involved in turning mature Schwann cells into the regenerative Schwann cell type after nerve injury (Arthur-Farraj et al., 2012). Overall, our gene expression data suggest that neural crest stem cell-characteristic genes are downregulated and Schwann cell/myelin-related transcripts are upregulated with progressing differentiation.

The notion that hEPI-NCSC-derived Schwann cells are functionally active is supported by our observation of close alignment and interactions of the Schwann cells with neurites in co-culture, as shown by confocal and electron microscopy. Schwann cells exhibited multiple protrusions that grew towards bundles of neurites and, in some cases, very close membrane-membrane associations were formed between the Schwann cells and neurites. The latter is considered an initial stage of myelination. There are no convincing reports in the literature on the generation of myelinating human Schwann cells in human Schwann cell-rodent DRG co-cultures.

Schwann cell differentiation was successful only in the continuous presence of SB431542, which is a selective and potent inhibitor of ALK4, 5 and 7 (ACVR1B, TGFBR1 and ACVR1C). SB431542 inhibits endogenous activin and TGFβ signalling without affecting more divergent BMP signalling that utilises ALK1, 2, 3 and 6 (ACVRL1, ACVR1, BMPR1A and BMPR1B) (Laping et al., 2002). Most likely, inclusion of SB431542 was necessary to guide stem cells away from the neuronal cell lineage, to inhibit TGFβ-related apoptosis early in Schwann cell differentiation and to avoid inhibition of SOX10 and P0 expression by TGFβ signalling (Morgan et al., 1994; Jessen and Mirsky, 2005; Kelsh, 2006). The βME/RA protocol for Schwann cell differentiation developed by Dezawa et al. (2001) for rat marrow stromal cells has also successfully been used with rat adipose-derived stem cells (Kingham et al., 2007), but was not applicable to hEPI-NCSC. RA treatment for 3 or 5 days (data not shown) was not conducive to Schwann cell differentiation. However, when RA was present throughout the culture period Schwann cell differentiation proceeded efficiently. The reason for this discrepancy between rodent and human cells is not obvious, as RA affects many signalling pathways that regulate cell differentiation. Alternatively, prolonged RA treatment might be required because depletion of the hEPI-NCSC pool occurs over a prolonged period of time in culture only due to continued self-renewal.

Notably, hEPI-NCSC-derived Schwann cells expressed transcripts for neurotrophic factors that are essential for nerve regeneration, and are expressed by Schwann cells *in vivo* during nerve regeneration, including NGF (Rush, 1984), NT-4/5 (Funakoshi et al., 1993), BDNF (Meyer et al., 1992), FGF2 (Ornitz and Itoh, 2001), FGF5 (McGeachie et al., 2001), LIF (Kurek et al., 1996) and IL6 (Bolin et al., 1995). Another important aspect for successful nerve regeneration includes cues from Schwann cells that promote angiogenesis in the injured microenvironment. VEGFA is a key angiogenic factor expressed in hEPI-NCSC, and *VEGFA* transcripts were upregulated sixfold in hEPI-NCSC-derived Schwann cells. It is thus likely that hEPI-NCSC-derived Schwann cells will contribute to angiogenesis *in vivo*. This notion is strongly supported by our observations that mouse EPI-NCSC (mEPI-NCSC; Hu et al., 2010) and cEPI-NCSC (McMahill et al., 2015) promote angiogenesis in the spinal cord injury of mice and dogs.

hEPI-NCSC are isolated directly from bulge explants as highly pure populations of migrating multipotent neural crest-derived stem cells without the need for purification or further manipulation, and within a short period of time. Krause et al. (2014) reported that skin-derived precursor cells (SKPs) from rodents and from human foreskin could generate myelinating Schwann cells. The method used by Krause et al. (2014) differs from ours in that it involves time-consuming isolation and purification procedures, as pieces of dermis had to be enzymatically digested, manually dissociated by pipetting, strained, and cultured to generate primary spheres. Primary spheres were subsequently treated with collagenase, followed by additional treatments, and finally placed into culture to generate secondary spheres, which were subsequently used for cell differentiation.

What is the Schwann cell subtype of the hEPI-NCSC-derived human Schwann cells? According to marker expression (high KROX20, low P0 expression in the absence of neurites) the hEPI-NCSC-derived Schwann cells could be categorised either as myelinating immature Schwann cells or as Remak cells (non-myelinating Schwann cells). However, KROX20 is specific for myelinating Schwann cells (Allodi et al., 2012) and was expressed throughout the 30-day culture period. P0 immunoreactivity increased over time in culture and was intense in co-culture with neurons. In the protocol presented here, immunoreactivity for GFAP, which is a marker for non-myelinating and immature Schwann cells (Allodi et al., 2012), was not detected initially and at low levels only towards the end of the 30-day culture period. Expression of p75NTR was strong initially but decreased with advancing *in vitro* differentiation. In addition to being a neural crest stem cell marker, p75NTR expression can also be indicative of non-myelinating Schwann cells or immature myelinating Schwann cells (Allodi et al., 2012). Taken together, at the end of the 30-day culture period, hEPI-NCSC-derived Schwann cells expressed markers for myelinating Schwann cells (KROX20, MBP, P0, S100B) as well as markers characteristic for immature Schwann cells and non-myelinating Schwann cells, that is (low) p75NTR immunofluorescence and (low) GFAP immunofluorescence, as well as *JUN* transcripts. Overall, the best fit for a Schwann cell subtype in the current culture conditions is an immature myelinating type Schwann cell. Culturing of the cells under standard myelinating and non-myelination culture conditions would further clarify this issue.

Taken together, hEPI-NCSC are multipotent human adult stem cells that are readily accessible in the bulge of hair follicles. Owing to their migratory ability, hEPI-NCSC can be isolated as a highly pure population of multipotent stem cells, and they can be expanded rapidly *ex vivo*. In this study, we demonstrated that hEPI-NCSC can be differentiated into human Schwann cells rapidly, with high efficiency and without the need for genetic manipulation or purification. hEPI-NCSC-derived Schwann cells express markers of myelinating Schwann cells that are conducive to nerve repair. For these reasons combined, hEPI-NCSC-derived human Schwann cells promise to be a valuable resource for translational research.

## MATERIALS AND METHODS

### hEPI-NCSC cultures

De-identified hairy skin biopsies were obtained with ethical approval (REC REF: 08/H0907/1) and hEPI-NCSC were isolated from human hair follicles exactly as described previously (Clewes et al., 2011). Briefly, anagen hair follicles were dissected and the bulge section placed into adherent culture. hEPI-NCSC emigrating from bulge explants were subcultured, expanded for 6 days exactly as described (Clewes et al., 2011) and cryopreserved.

### Differentiation of hEPI-NCSC into Schwann cells

Cryopreserved hEPI-NCSC were thawed, seeded at 2500-5000 cells per CELLstart-coated 35-mm culture plate and cultured at 36.8°C, 5% CO_2_ and 5% O_2_ for several days. At differentiation day minus 3 (D−3), cells in XP medium were treated with 500 ng/ml recombinant human (rh) sonic hedgehog (C24II) N-terminus (rhSHH) and 500 nM CHIR99021 for 2 days. At D–1, the cells were subcultured into XP medium at a density of 30,000 cells per 35-mm plate and 1000 cells per poly-D-lysine- and laminin-coated 13-mm diameter glass coverslip. The next day (D0), cells were cultured in Alpha-modified MEM in the presence of 10% FBS and 1 mM βME for 24 h, which was followed by treatment with 35 ng/ml all-trans RA according to the method of Dezawa et al. (2001). At D3, the FBS concentration was reduced to 1%. Starting at D0, 10 µM SB431542 was also added to the culture medium. RA and SB431542 remained in the culture medium throughout the culture period. At D3, 10 ng/ml rhFGF2, 5 ng/ml rhPDGF-BB, 5 µM forskolin and 200 ng/ml rhNRG1 were added to the culture medium. The culture medium was exchanged every 48 h. For details of equipment and reagents see supplementary material Table S1.

### DRG explant culture

‘Green’ mice [C57BL/6-TgN(ACTbEGFP)1Osb; Okabe et al., 1997] and wild-type Wistar rats were handled in accordance with protocols approved by the Animal Care Committee of Newcastle University (project licence 60/3876). Whole-mount DRG explants were dissected from E15.5 Wistar rat embryos (Päiväläinen et al., 2008; Liu et al., 2011). Dissociated DRG cultures were prepared from adult Green mice (Malin et al., 2007; Johnson et al., 2001). To remove non-neuronal cells, DRG explants were treated with camptothecin (20 µM) three times for 48 h each, on D2-D5, D6-D8 and D13-D15.

### Co-cultures of hEPI-NCSC-derived Schwann cells and DRG neurons

At D28, hEPI-NCSC-derived Schwann cells were detached with TrypLE and seeded onto camptothecin-treated DRG explants on D17 of DRG explant culture or D10 of dissociated DRG neuron culture, at 12,000 Schwann cells per 13-mm coverslip, and incubated for 21 days in co-culture medium consisting of alpha-modified MEM, penicillin/streptomycin, B27 supplement without RA, 5 ng/ml rhPDGF-BB, 5 µM forskolin, 10 ng/ml rhNRG1, 50 µg/ml ascorbic acid, 20 ng/ml rhPDGF, 20 ng/ml GDNF, 20 ng/ml rhβNGF, 20 ng/ml rhNT3 and 10 µM SB431542. For details of reagents see supplementary material Table S1.

### Indirect immunocytochemistry

Indirect immunocytochemistry was performed exactly as described previously (Clewes et al., 2011; Narytnyk et al., 2014a,b). Briefly, cultures were fixed (4% paraformaldehyde), rinsed, blocked, and incubated overnight with primary antibodies in 0.1% Triton X-100 in the cold. Subsequently, cultures were rinsed, incubated with secondary antibodies, rinsed and mounted with Vectashield hard set mounting medium with DAPI. Fluorescence was observed with an Axio Imager Z1 fluorescence microscope or with a Nikon Eclipse Ti confocal microscope. Secondary antibodies were designed for multiple labelling with minimal cross-reactivity with human, bovine, horse, rabbit/mouse and swine serum proteins. Negative controls did not show immunofluorescence. Data were statistically analysed using Dunnett's test. Primary and secondary antibodies are detailed in supplementary material Table S1.

### Electron microscopy

Co-cultures of hEPI-NCSC-derived Schwann cells with rat DRG explants were fixed for electron microscopy with 2.5% glutaraldehyde in 0.1 M Sorenson's phosphate buffer for 1 h at room temperature, rinsed with Sorenson's phosphate buffer, the DRG in the centre of the co-culture removed leaving axons and Schwann cells attached to the culture well, well bottoms isolated, shipped in buffer at room temperature and processed for electron microscopy as follows: post-fixation in 1% osmium tetroxide in PBS, dehydration in a graded series of ethanol, embedding in Epon resin and polymerisation for 48 h at 60°C. The region of interest was identified using a light microscope, cut out with a single-edge razor blade, flat-embedded in Epon resin and polymerised as before. Ultrathin sections of 70 nm were cut with a Diatome diamond knife, mounted on pioloform/carbon films on copper slot grids and counterstained with 4% uranyl acetate and Reynolds’ lead citrate. Images were taken using a JEM 1400 transmission electron microscope equipped with an AMT XR60 digital camera.

### RNA isolation, gene expression profiling and quantitative real-time PCR (qPCR)

Total RNA was extracted from snap-frozen homogenised cells at D−3 and D21, and RNA purity was determined using an Agilent 2100 Bioanalyser [RNA integrity number (RIN) values: D−3, 9.9; D21, 10.0]. Gene expression profiling was performed by the Genome Centre at Barts and The London School of Medicine and Dentistry using a Human HT-12 v4 array and the Illumina platform. Data have been deposited in the NCBI Gene Expression Omnibus with accession number GSE61273. For qPCR, 1 µg total RNA was incubated with DNaseI and processed exactly as described previously (Clewes et al., 2011). Ct values for targets were normalised to the average Ct value of *GAPDH*. Fold changes in expression were calculated using the ΔΔCt method. Primers are listed in supplementary material Table S1.

## Supplementary Material

Supplementary information
