# Supplementary material for: Human epidermal neural crest stem cells as a source of Schwann cells
Source: Development. 2015 Sep 15;142(18):3188–97. doi: 10.1242/dev.123034 (PMC4582175; doi:10.1242/dev.123034)
Supplement: Supplementary information [file supp_142_18_3188__index.html]

Supplementary information 

# Human epidermal neural crest stem cells as a source of Schwann cells

## DEV123034 Supplementary information

- Supplementary information
