# Supplementary material for: Human epidermal neural crest stem cells as a source of Schwann cells
Source: Development. 2015 Sep 15;142(18):3188–97. doi: 10.1242/dev.123034 (PMC4582175; doi:10.1242/dev.123034)
Supplement: Supplementary information [file supp_dev.123034_DEV123034supp.pdf]

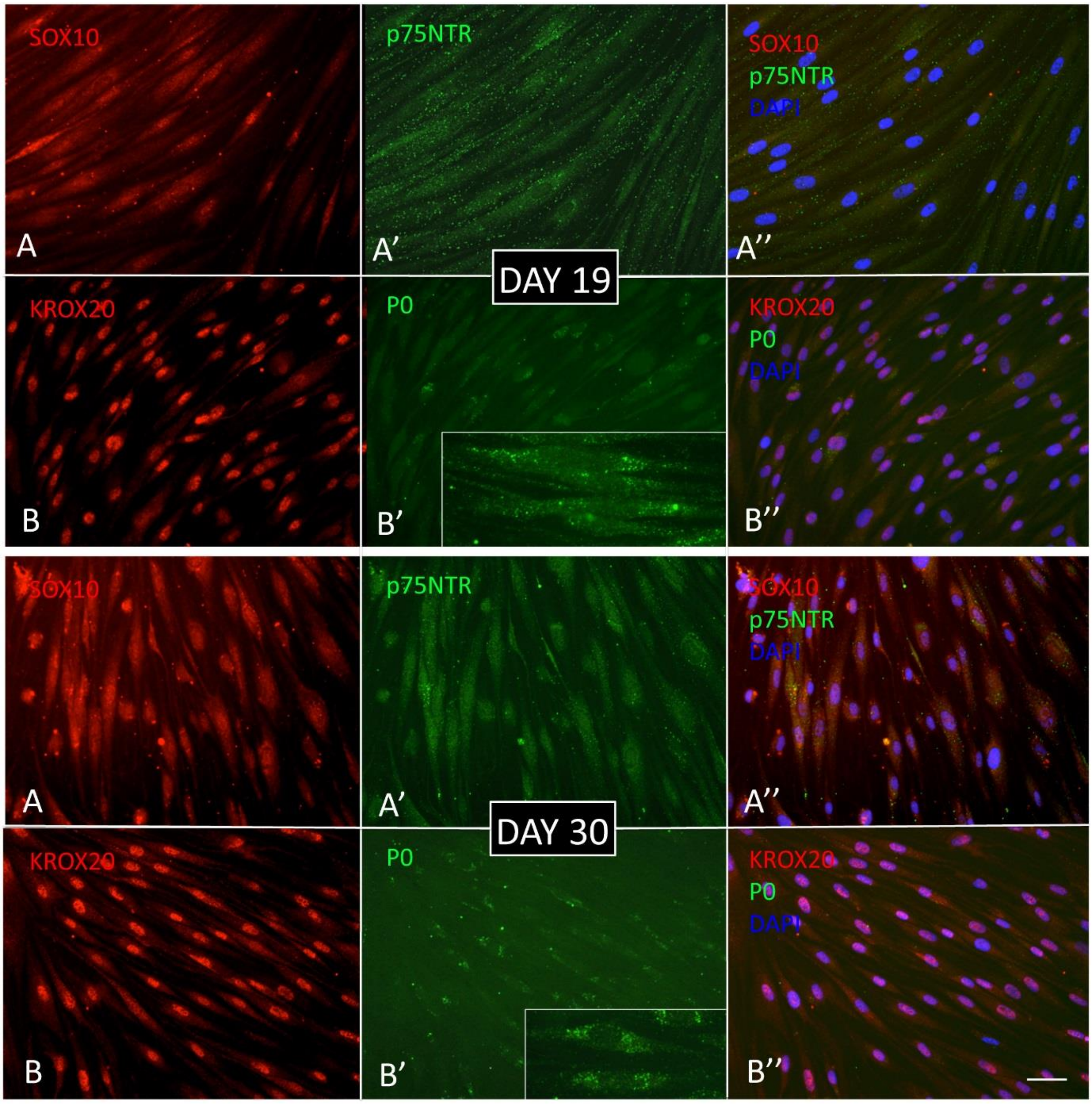

Figure S1: **Immunocytochemistry of Schwann cell marker expression at late stages of in vitro differentiation.** ***Day 19:*** (A) SOX10 immunoreactivity in all cells, mostly cytoplasmic. (A’) very low p75NTR expression in same area;. (A’’) SOX10/p75NTR/DAPI merged images. (B) Nuclear KROX20 expression in all cells. (B’) Increased P0 immunoreactivity in same area as in A. (B’’) KROX20/p75NTR/DAPI merged images. ***Day 30:*** (A) SOX10 immunoreactivity in all cells, nuclear and cytoplasmic. (A’) Low p75NTR immunoreactivity; same area as in A. (A’’) SOX10/p75NTR/DAPI merged images. (B) KROX20 immunoreactivity in all cells; nuclear localisation; (B’) Increased P0 immunoreactivity in all cells; inset, two cells at higher magnification. (B’’) KROX20/P0/DAPI merged images. Day 19 and Day 30, A-B’’, bar, 50 μm.

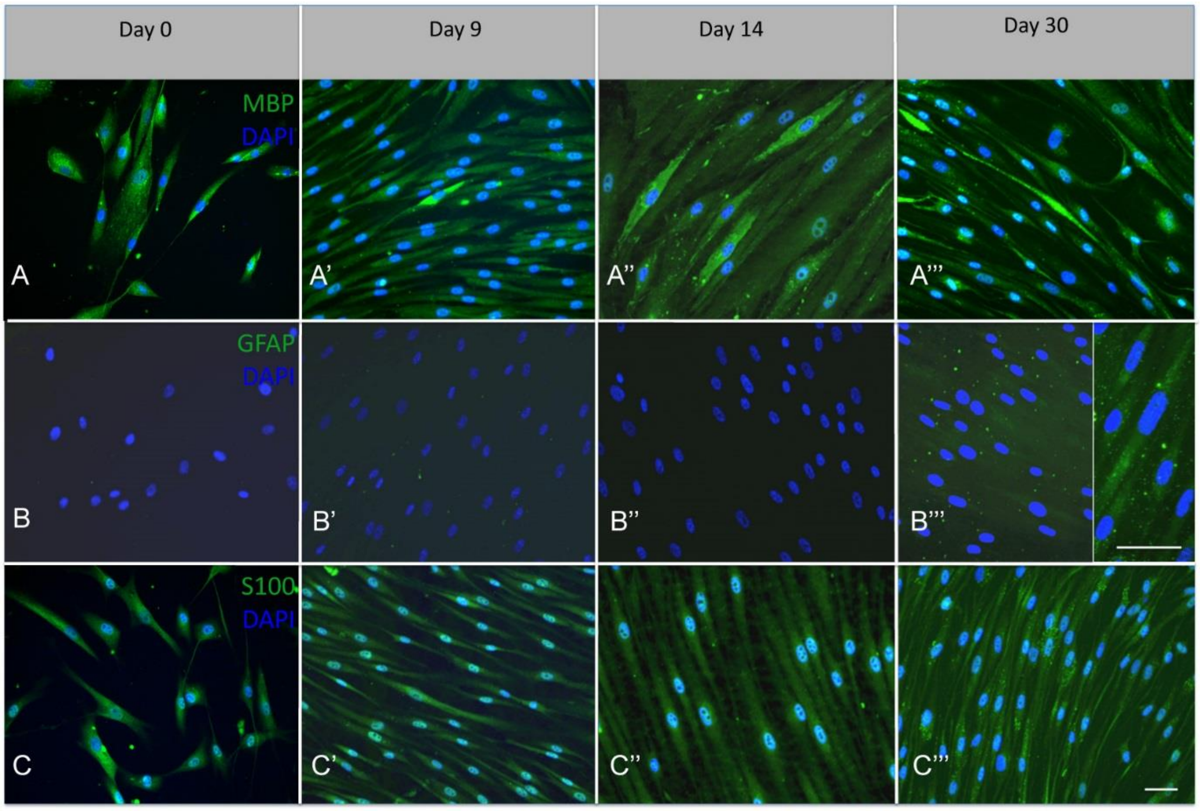

Figure S2: **Immunocytochemistry of MBP, GFAP and S100 expression during in vitro development.** Marker (green) and DAPI nuclear stain (blue) merged images. (A - A''') Myelin basic protein (MBP) immunoreactivity on D0 (A), D9 (A'), D14 (A'') and D30 (A'''); all cells are immunopositive throughout in vitro differentiation. (B - B''') Glial fibrillary acidic protein (GFAP) immunoreactivity on D0 (B), 9 (B'), D14 (B'') and D30 (B'''); B''' inset, 6 cells at higher magnification. There was not detectable GFAP immunoreactivity until the end of the 30-day culture period, at which time GFAP immunoreactivity was at barely detectable levels (B'''). (C – C''') S100/DAPI merged images from culture at D0 (C), D9 (C'), D14 (C'') and D30 (C'''). All cells showed strong S100 immunoreactivity throughout the culture period. A-C''', bar, 50 µm. Inset, bar 50 µm.

Development • Supplementary information

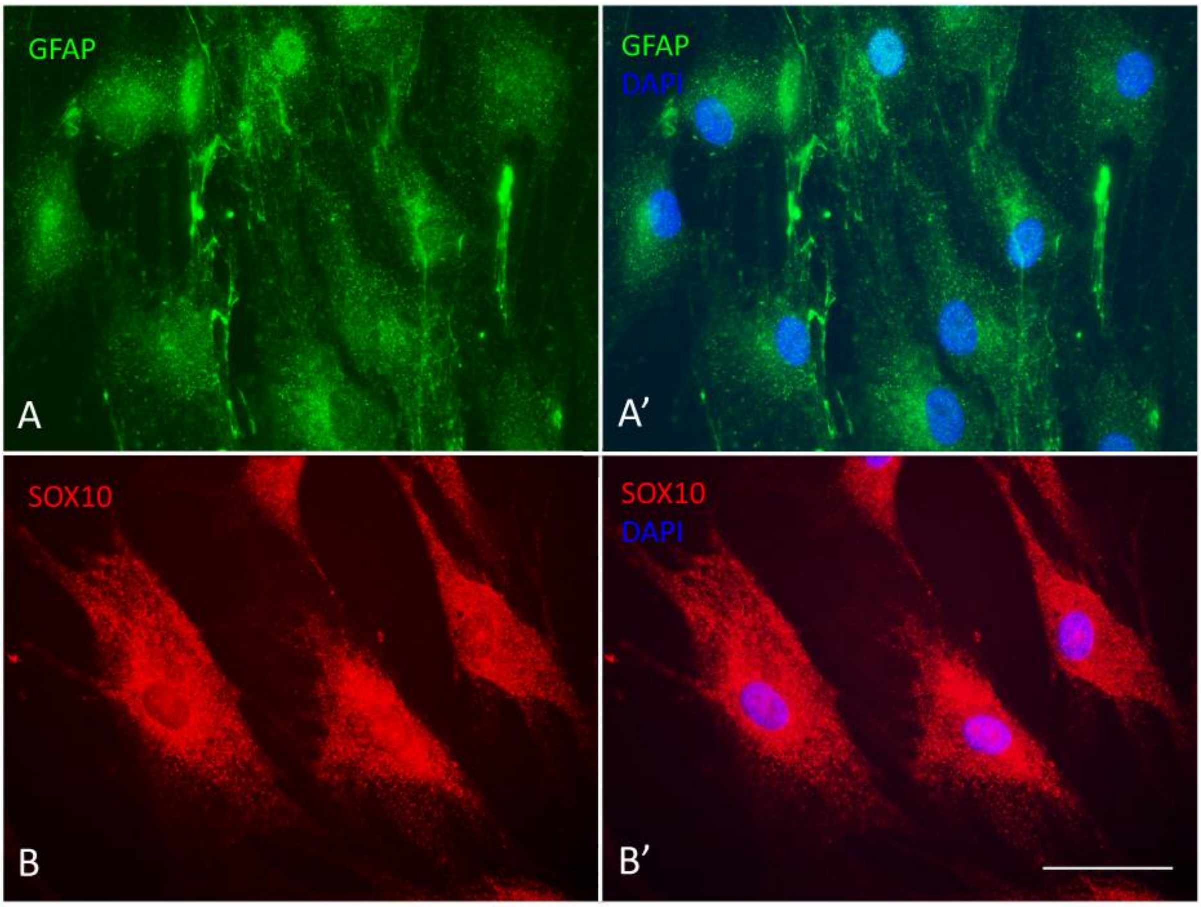

Figure S3: GFAP immunoreactivity in alternative culture conditions. (A) In the absence of SHH, CHIR99021 and retinoic acid, highly intense GFAP immunoreactivity and (B) intense SOX10 cytoplasmic immunoreacitivy is observed. A’, B’ merged images with DAPI nuclear stain. A-B’, bar, 50 µm.

Click here to Download Figure S4

Figure S4: Heat map with cluster analysis dendrograms. The heat map is presented using the log 10 of the average signal.

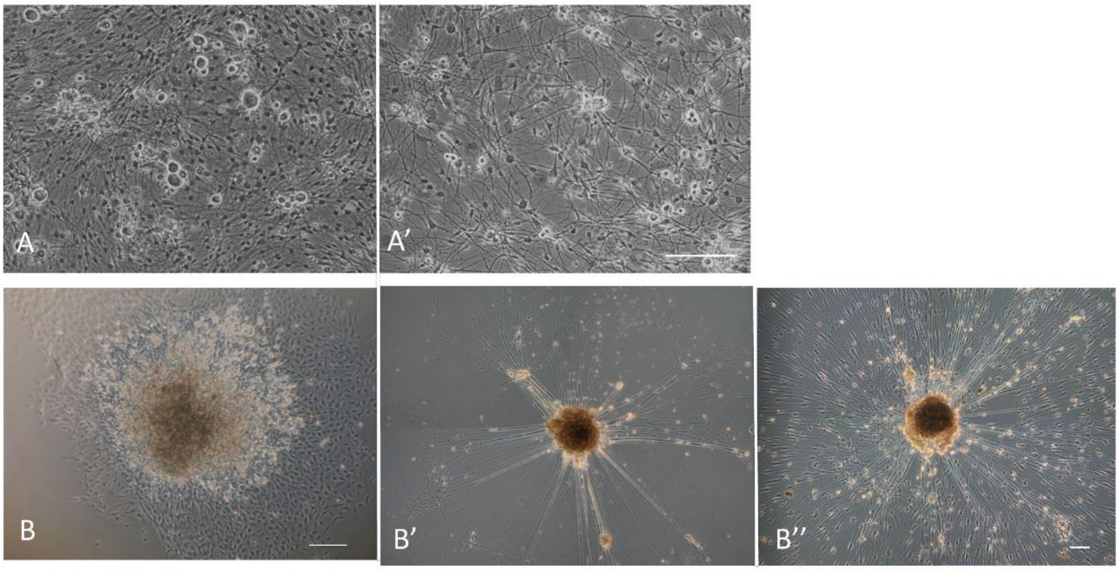

Figure S5: **Dorsal root ganglion (DRG) explant cultures.** DRG cultures were either dissociated (A, A') or plated as non-dissociated ganglia (B – B'';). (A) Adult mouse embryo dissociated DRGs without camptothecin treatment. Many large and small diameter sensory neurons are visible, as well as numerous non-neuronal cells (A') Adult mouse embryo dissociated DRG at Day 9 post-explantation after camptothecin treatment; non-neuronal cells and large diameter sensory neurons absent due to camptothecin toxicity. (B) E15.5 rat embryo whole DRG explant at 24 hours post-explantation in the absence of camptothecin with numerous emigrating non-neuronal cells. (B') E15.5 rat embryo DRG after camptothecin treatment at Day 21 post-explantation; absence of non-neuronal cells. (B'') DRG explant after camptothecin treatment in the presence of hEPI-NCSC derived Schwann cells aligned with neurites radiating from the DRG explant at 38 Days post-explantation (21 Days of co-culture). A, A', bar 200 µm. B, bar 200 µm; B'B'', bar, 200 µm.

Table S1. Equipment, reagents and their sources

Equipment

Class II safety hood (Clean Air, Clean Air Techniek, Woerden, Netherlands)

Confocal microscope (Nikon A1R confocal microscope system, Nikon UK, Kingston Upon Thames, UK)

Digital camera powerShot (A650IS, Canon, Tokyo, Japan)

Dissecting microscope (Stemi 2000, Carl Zeiss UK, Cambridge, UK)

Fluorescence microscope (Imager.Z1, Carl Zeiss UK)

Illumina platform

Inverted microscope (Axiovert 40C, Carl Zeiss UK)

Taqman 7500 thermocycler (Applied Biosystems, Foster City, CA)

Transmission electron microscope

Two-gas incubator at 5% CO_2 and 5% O_2 (CO_2 incubator with O_2 control, BINDER, Tuttlingen Germany)

Reagents

13-mm in diameter round cover glasses (VWR, Leicestershire, UK, Cat# 631-0149)

1X SITE+3 liquid medium supplement (Sigma, Poole, UK, Cat# S5295)

2-mercaptoethanol (2ME) (Sigma, Cat# M3148)

24 multi well plates (VWR, Cat# 734-2325)

35-mm culture plate (Fisher Scientific UK, Leicestershire, UK, Cat# 430165)

All-trans-retinoic acid (RA) (Sigma, Cat# R2625)

Alpha MEM with UltraGlutamine™ I, deoxyribonucleoside and ribonucleosides (Lonza, Basel, Switzerland, Cat# BE02-002F)

Ascorbic acid (Sigma, Cat# A4544)

B27 supplement minus vitamin A (Life Technologies, Paisley, UK, Cat # 12587-010)

CELLstart (Life Technologies, Cat# A10142-01)

CHIR99021 (Stemgent, Cambridge, MA, Cat# 04-0010)

Camptothecin (Millipore, Watford, UK, Cat# 208925)

Development • Supplementary information

Collagenase I (Sigma, Cat# C5138)

DNaseI (Life Technologies, Cat# 18068-015)

Dulbecco's phosphate buffer saline without Ca^{2+} and Mg^{2+} (DPBS (+), Life Technologies, Cat# 14190-094)

Fast SYBR Green Master mix (Applied Biosystems, Cat# 4385612)

Fetal bovine serum (HyClone, Thermo Fisher, Cramlington, UK, Cat# SH30070.02)

Forskolin (Sigma, Cat# F6886)

GlutaMAX-I (Life Technologies, Cat# 35050-038)

Glutaraldehyde, 25% EM Grade vacuum distilled (Agar Scientific, Elektron Technology, Essex, UK, Cat# AGR1010)

Lab-Tek 4-well chamber glass slides (Fisher Scientific UK, Cat# 177399),

Laminin (Sigma, Cat# L2020)

Matrigel, basement membrane matrix, growth factor reduced (DB Biosciences, Bedford USA, Cat# 356230)

Na_2HPO_4 (Sigma, Cat# S7907)

$NaH_2PO_4 \cdot H_2O$ (Sigma, Cat# 71507)

NeuroCult XF basal medium (Stem Cell Technologies, Grenoble, France, Cat# 05761)

NeuroCult-XF Proliferation Supplements, (Stem Cell Technologies, Cat# 05763)

Normal goat serum (NGS) (Sigma, Cat# G9023)

Oligo(dT)$_{20}$ primer (Life Technologies, Cat# 18418-020)

Paraformaldehyde (PFA) (Sigma, Cat# 158127)

Penicillin/streptomycin (Sigma, Cat# P0781)

Phosphate buffer saline without Ca^{2+} and Mg^{2+} (PBS) (Life Technologies, Cat# 20012)

Poly-D-lysine (Sigma, Cat# P6407)

Recombinant human brain-derived neurotrophic factor (rhBDNF) (R&D Systems, Abingdon, UK, Cat# 248-BD/CF)

Recombinant human epidermal growth factor (rhEGF) (Stem Cell Technologies, Cat# 02653)

Recombinant human fibroblast growth factor 2 (rhFGF2) (R&D Systems, Cat# 233-FB)

Recombinant human glial cell line-derived growth factor (rhGDNF) (R&D Systems, Cat# 212-GD/CF)

Development • Supplementary information

Recombinant human neuregulin-1 (rhNRG) (R&D Systems, Cat# 377-HB/CF)

Recombinant human neurotrophin 3 (rhNT3) (R&D Systems, Cat# 267-N3/CF)

Recombinant human platelet-derived growth factor (PDGF)-BB (rhPDGF-BB) (R&D Systems, Cat# 220-BB)

Recombinant human sonic hedgehog (C24II) N-terminus (rhSHH) (R&D Systems, Cat# 1845-SH/CF)

Recombinant human β-nerve growth factor (rhβNGF) (R&D Systems, Cat# 256-GF/CF)

SB431542 (Stemgent, Cat# 04-0010)

SuperScript III Reverse transcriptase (Life Technologies, Cat# 18080-044)

Triton X-100 (Sigma, Cat# T8787)

TrypLE select (Life Technologies, Cat# 12563-011)

Vectashield hard set mounting medium with 4',6-diamidino-2-phenylindole (DAPI) (Vector Laboratories, Peterborough, UK, Cat# H-1500)

dNTP mix (Life Technologies, Cat# 18427-088)

Primary Antibodies

Rabbit anti-myelin protein zero (P0) antibody (1:100) (Abcam, Cambridge, UK, Cat# ab31851)

Rabbit anti-myelin basic protein (MBP) antibody (1:400) (Abcam, Cat# ab124493)

Rabbit anti-S100 antibody (1:200) (Dako UK, Ely, UK, Cat# Z0311)

Rabbit anti-glial fibrillary acidic protein (GFAP) (1:200) (Abcam, Cat# ab68428)

Rabbit anti-p75NTR antibody (1:500) (Millipore, Cat# ab1281)

Goat anti-SOX10 antibody (1:100) (R&D, Cat# AF2864)

Mouse anti-early growth response 2 (KROX20) (1:100) antibody (Abcam, Cat# 156765)

Mouse anti-human nuclei antibody (1:200) (Millipore, Cat# MAB1281)

Chicken anti-neurofilament H antibody (1:10,000) (Abcam, Cat# ab4680)

Secondary antibodies

All secondary antibodies were purchased from Stratech Scientific, Newmarket, UK

Alexafluor-488 conjugated goat anti-rabbit IgG (Cat# 111-545-144)

Alexafluor-594 conjugated donkey anti-goat IgG (Cat# 705-585-147)

Alexafluor-488 conjugated donkey anti-rabbit IgG (Cat# 711-545-152)

Alexafluor-594 conjugated goat anti-rabbit IgG (Cat# 111-585-144)

Cy5 conjugated goat anti-mouse IgG (Cat# 115-175-146)

Alexafluor-488 conjugated Goat anti-chicken IgY (Cat# 103-545-155)

Gene-specific RT2 qPCR primers

All primers are from Qiagen, Manchester, UK

SOX10 (Cat# PPH02458A)

P75 (Cat# PPH00821A)

GFAP (Cat# PPH02408E)

GAPDH (Cat# PPH00150E)
